# Early diagnosis and follow-up of cerebrotendinous xanthomatosis in infant siblings presenting with congenital diarrhea: A case study from Saudi Arabia

**DOI:** 10.1016/j.ymgmr.2025.101188

**Published:** 2025-01-13

**Authors:** Badr Mohammad Alsaleem, Amna Basheer Ahmed, Muhannad M. Alruwaithi, Tarig Yassin Alamery, Norah Nasser Alrajhi

**Affiliations:** a Pediatric Gastroenterology Section, Intestinal Failure Program, Children's Hospital, King Fahad Medical City, Riyadh Second Health Cluster, Riyadh 11525, Saudi Arabia; b Pediatric Department, South Al Qunfudah General Hospital, Al Qunfudah 28821, Saudi Arabia

**Keywords:** Cerebrotendinous xanthomatosis, Diarrhea, Cholestasis, Cataracts, Mental retardation

## Abstract

Cerebrotendinous xanthomatosis (CTX) is a rare autosomal recessive neurometabolic genetic disease resulting from defects in the bile acid metabolism. This report describes cases diagnosed with CTX at an exceptionally early age – 4 months (Patient #2 and #3) – making them the youngest reported cases to date. All three presented with intractable congenital diarrhea, a hallmark manifestation of the disease. The diagnosis was confirmed through metabolic bile acids analysis in urine and genetic testing. The siblings were treated with Chenodeoxycholic acid (15 mg/kg/day) during the first year of treatment, resulting in an improvement in diarrhea in all three. However, cognitive function remained unimproved in one patient. Additionally, the presence of dysmorphic features, observed in these patients, have not been documented in previous CTX cases. The diagnosis prompted solely by the persistent diarrhea, highlights a critical, under-recognized early manifestation. These findings underscore the importance of raising awareness among physicians to enable early diagnosis and timely treatment, which may prevent disease progression.

## Highlights

• CTX is a rare, treatable genetic disease with early symptoms of diarrhea and cataracts.
• Early CDCA treatment improves symptoms and prevents disease progression.
• Detailed patient history and awareness among physicians is necessary for early diagnosis.

## 1 Introduction

Cerebrotendinous xanthomatosis (CTX; OMIM#213700) is a rare, autosomal recessive, neurometabolic, genetic disorder that is treatable. It was first described in 1937 by Van Bogaert and his colleagues as a disease that presents with tuberous and tendon xanthomas, premature cataracts, and discrete neurological symptoms affecting both the central as well as peripheral nervous systems. The mutation in the cytochrome P450 *CYP27A1* gene (OMIM*606530) results in defective production of sterol 27-hydroxylase enzyme that converts cholesterol to chenodeoxycholic acid (CDCA) and cholic acids leading to impaired bile acid synthesis [[1], [2], [3], [4], [5], [6]]. CTX is associated with abnormally high levels of cholestanol, other sterols, and stanols in the blood. Additionally, there is a buildup of cholestanol and cholesterol in the brain, tendon xanthomas, and bile. Reduced levels of CDCA impair the feedback regulation of cholesterol 7-alpha-hydroxylase leading to the formation of cholestanol. This is a rate-limiting step in the bile acid synthesis. Furthermore, the excretion of cholestanol, a metabolite of cholesterol is impossible. As a result, it accumulates in the plasma and gets deposited in various lipophilic tissues, such as the eyes and cerebellum in the brain, causing cataracts; in tendons, leading to tendon xanthomas, and in the nervous system, resulting in neurological dysfunctions [1,2]. Moreover, the excretion of bile alcohol is markedly increased in the bile, feces, and urine. It is believed that persistent diarrhea, which is commonly observed in younger children, is caused by a bile acid deficit. Chronic childhood diarrhea is one of the first and most commonly reported clinical manifestations observed in CTX and recognized since 1991 [[7], [8], [9]].

The prominent clinical presentations of CTX include persistent diarrhea, tendon xanthomas, bilateral cataract formation, and neurological impairments such as cerebellar ataxia, peripheral neuropathy, seizures, pyramidal and extrapyramidal signs, cognitive impairments, behavioral and psychiatric disturbances, and dementia. In addition, premature atherosclerosis, osteoporosis, and infantile cholestasis have also been reported in some cases [2,5,[8], [9], [10], [11]].

The disease diagnosis is based on the elevated serum cholestanol level and the determination of urinary bile acids metabolites, which show high bile acid alcohols and low chenodeoxycholic acid levels. Therefore, CDCA is currently being used as a standard of care treatment. CDCA can help achieve normal levels of sterols, bile acids, bile alcohols, and cholestanol. When CDCA was administered during the early stages of the disease, it was found to prevent the adverse clinical manifestations of the disease from occurring or progressing [1,3]. CTX is considered a rare disorder; however, it often remains underdiagnosed or misdiagnosed due to its nonspecific signs and symptoms and overlap with other common ailments [7]. The signs, symptoms, severity, and age of onset vary significantly across patients. Hence, an awareness about the disease is highly important.

The early disease diagnosis of CTX is based on the most prominent clinical features, and its early treatment, especially at a young age, is highly desirable to prevent any potential neurological or systemic complications [1,3]. Unfortunately, most cases in the literature are diagnosed late, typically in the third decade of life, when neurological involvement becomes evident [2,4,7]. Therefore, the present case study aims to guide healthcare professionals in early diagnosis of CTX and its effective management from an early age to prevent any potential neurological and systemic disorders. We described and discussed the clinical and laboratory findings as well as the outcomes after treatment, in three siblings with CTX from Saudi Arabia.

## 2 Case reports

### 2.1 Patient 1

**Fig. 1:**
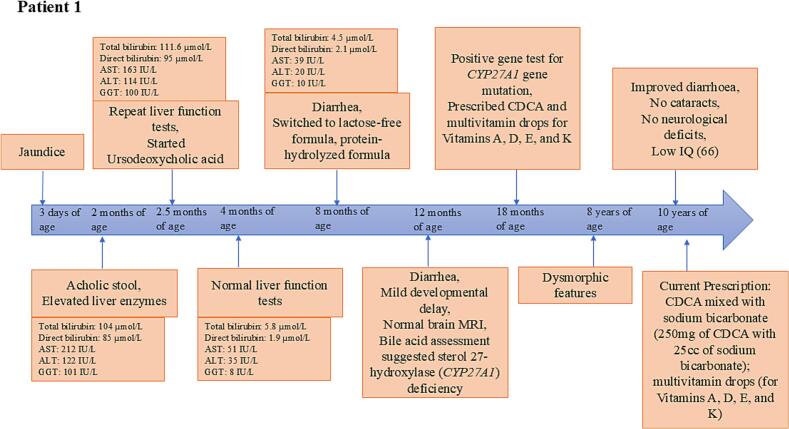
Timeline of symptoms and key biochemical test parameter for Patient 1. Figure 1 is shown to evaluate the timeline of Patient 1's history, from the age of 3 days to 10 years. It indicates the key symptoms, changes in the biochemical parameters over time, and the time of treatment initiation. AST: Aspartate aminotransferase; ALT: Alanine aminotransferase; GGT: Gamma-glutamyl transferase; IQ: Intelligence Quotient; CDCA: Chenodeoxycholic acid.

Patient 1 was a Saudi boy with a history of jaundice since the third day of his life. His birth weight was normal (3.25 kg), and he exhibited average growth and development without any respiratory difficulties. He was born to consanguineous parents without any family history of ophthalmological disorders, neurological conditions, or neonatal deaths. The patient first visited the hospital at two months of age. Diarrhea was absent at that time, however, acholic stools were reported. It is interesting to note here that, even though the onset of the disease first appeared on the third day of life, medical advice was obtained only in the second month. At two months of age, his physical examination showed a below-average z-score for growth parameters [weight: 3.84 kg (z-score: −2.08), height: 55 cm (z-score: −1.31)]. The z-score for all the instances was calculated based on PediTools - 2000 CDC growth charts [12]. The blood investigation at two months of age showed elevated levels of total and direct bilirubin along with an elevated liver enzyme profile. The coagulation profile and lipid parameters were within normal ranges. Repeat liver function tests after 2 weeks (at the age of 2.5 months), showed an increase in total and direct bilirubin and a slight decrease in liver enzymes. Fig. 1 is shown to evaluate the timeline of Patient 1's history.

The urine for bile acid metabolisms showed no defect in the cholesterol-bile acid biosynthetic pathway or any abnormality in peroxisomal beta-oxidation of bile acid intermediates. Abdominal ultrasound revealed normal liver echogenicity with a liver span of 7.3 cm, a common bile duct measuring about 1 mm, and an unremarkable gall bladder. The spleen showed normal echo texture, and both kidneys were normal.

The patient was started on Ursodeoxycholic acid and discharged. At four months of follow-up, bilirubin levels started to improve and resolve; liver function tests appeared normal. The coagulation profile was also normal. The patient continued with follow-up visits and the mother began weaning food at 6 months of age.

During the eight-month follow-up visit, the mother complained that the baby had diarrhea since the last two months. The diarrhea was watery, occurring 4–5 times a day, with more episodes toward the end of the day, and appeared oily without blood (Vesikari score: 5). He was on regular milk formula and a diet appropriate for his age. There was no history of vomiting, recurrent infections, skin rash, jaundice, itching, or previous admissions due to dehydration. There was no family history of a similar condition. His development was as expected for his age. On physical examination, growth parameters showed a below-average z-score for the growth parameters [weight: 7.2 kg (z-score: −1.85), height: 65 cm (z-score: −2.10)]. The patient was active without any signs of dehydration, jaundice, or dysmorphic features. The abdomen was soft with no organomegaly. Blood investigation showed white blood cells 13.5 × 10^9^/L, hemoglobin 14 g/dL, and platelets 558 × 10^9^/L. The celiac profile and sweat chloride tests were negative. Liver function test showed normal levels for AST (39 IU/L), ALT (20 IU/L), GGT (10 IU/L), total bilirubin (4.5 μmol/L), direct bilirubin (2.1 μmol*/*L), and Alkaline phosphatase (ALP) (236 IU/L). Vitamins A and E levels were also normal, but vitamin D was low at 38 nmol/L. Fecal elastase was normal (452 μ/g). Despite changing the formula to lactose-free and later to a protein-hydrolyzed formula, diarrhea did not improve. Upper endoscopy showed normal duodenal villi and no infiltrative cells.

At 12 months of age, mild developmental delay was noted, mainly in fine motor skills and speech (Based on the Denver Developmental Screening Test). Brain MRI appeared normal and signal intensity of cerebral and cerebellar hemispheres with no diffusion restriction or abnormal parenchymal-meningeal enhancement in the post-contrast images and normal gray-white matter differentiation. Bile acid metabolism assessment in urine showed an elevation of polyhydroxylated bile alcohol glucuronides, suggesting sterol 27-hydroxylase (*CYP27A1*) deficiency, a bile acid synthesis disorder. At the age of 18 months, exome-wide analysis, using Next Generation Sequencing (NGS) was performed. This resulted in the identification of a pathogenic mutation, CYP27A1; Chr2(GRCh37):g.219677818C > T; NM_000784.3:c.1016C > T (p.(Thr339Met)) homozygous. This nomenclature uses the HGVS mutation nomenclature guidelines. Before treatment, the cholestanol level reported was 31 mg/mL (Other cholestanol level data are not available due to resource limitations). The patient was prescribed CDCA (15 mg/kg/day) and multivitamin drops (for vitamins A, D, E, and K).

**Fig. 2:**
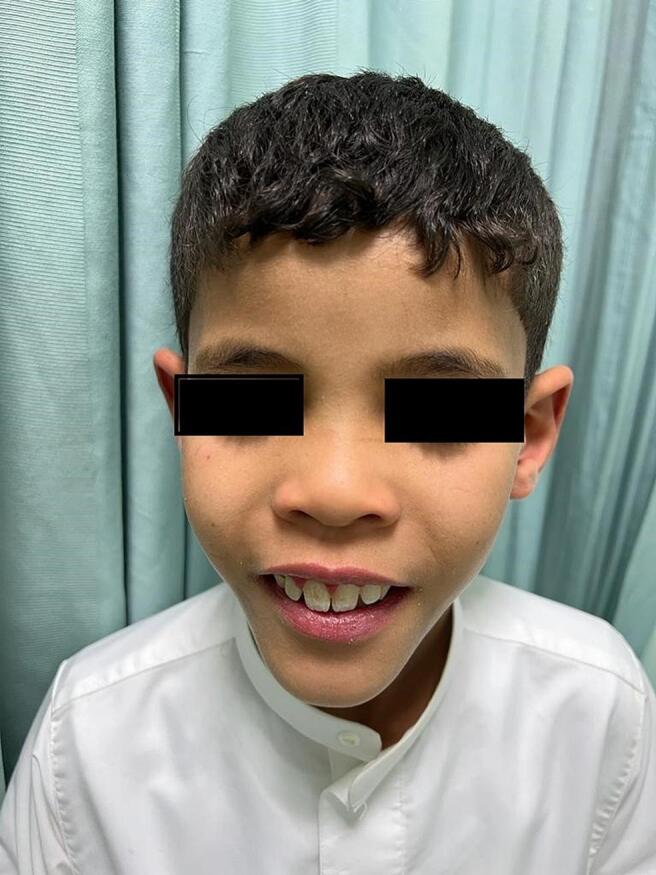
Dysmorphic features in Patient 1.

Around the age of 8 years, he developed some dysmorphic features, such as a broad nasal base, bulbous nose, long philtrum, prominent cheeks, and pointed chin, which had not been reported previously (Fig. 2). As of March 2024, the patient is 10 years old; his diarrhea has improved, and has not reported cataracts or neurological deficits. However, his cognitive function was low, and his IQ (intelligence quotient) was subnormal (IQ score: 66; normal average score for IQ is 100), hence, he was enrolled in a special school. Eight months ago, CDCA was prescribed which was to be mixed with sodium bicarbonate (NaHCO3) (250 mg of CDCA with 25 cc of sodium bicarbonate).

During the last visit to the clinic (at the age of 10 years), the patient showed increased awareness and attention, which the parents also noted. He was referred to the Mental Health Department for further evaluation and assessment.

### 2.2 Patient 2

**Fig. 3:**
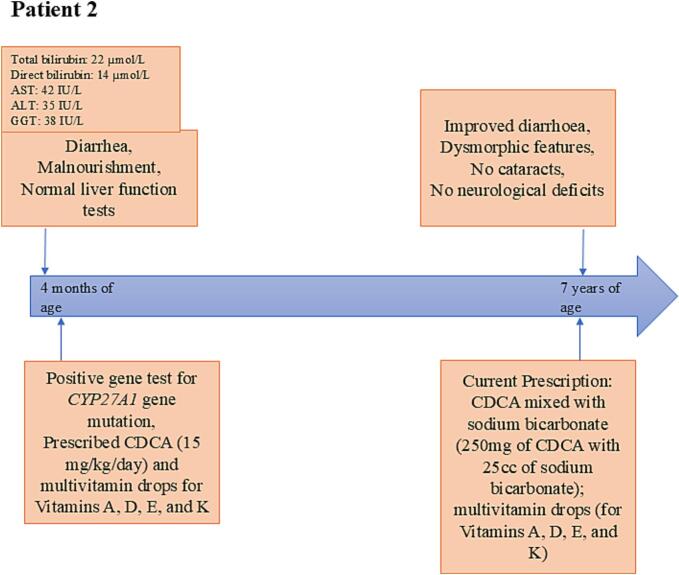
Timeline of symptoms and key biochemical test parameter for Patient 2. Figure 3 shows the key symptoms of CTX and biochemical parameters observed in the life of Patient 2, from the age of 4 months to 7 years. AST: Aspartate aminotransferase; ALT: Alanine aminotransferase; GGT: Gamma-glutamyl transferase; IQ: Intelligence Quotient; CDCA: Chenodeoxycholic acid.

Patient 2 was a girl child, the sister of Patient 1 (Index patient) who first visited the clinic with her mother at the age of 4 months with a chief complaint of diarrhea and signs of malnourishment. At the time of the first visit, the diarrhea score was 5, per the Vesikari score system; and her physical examination showed a lower z-score than the average [weight: 4.8 kg (z-score: −1.88), height: 56 cm (z-score: −2.22)]. Her CBC and electrolytes were normal. Her liver function tests were also within the normal range – ALT: 35 IU/L; AST: 42 IU/L; and GGT: 38 IU/L. However, levels of total bilirubin (22 μmol/L; normal range: 5.1 - 17 μmol/L) and direct bilirubin (14 μmol/L; normal range: 1.7–5.1 μmol/L) were elevated. (Fig. 3). Apart from these, laboratory tests for coagulation disorders were also conducted which reported Prothrombin Time (PT) of 12 s (normal range: 11 to 13.5 s), international normalized ratio (INR) of 1.1 (normal range: 0.8 to 1.1); and Partial Thromboplastin Time (PTT) of 33 s (normal range: 33.3 to 56 s).

**Fig. 4:**
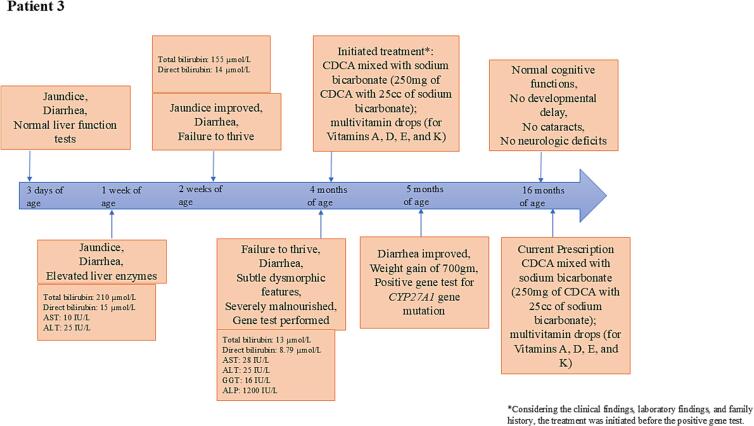
Timeline of symptoms and key biochemical test parameter for Patient 3. Figure 4 shows the key symptoms of CTX that appeared in the life of Patient 3, from the age of 3 days to 16 months. It also indicates the changes in the biochemical parameters over time and when the treatment was initiated. AST: Aspartate aminotransferase; ALT: Alanine aminotransferase; GGT: Gamma-glutamyl transferase; ALP: alkaline phosphatase; CDCA: Chenodeoxycholic acid.

Keeping in mind the history of Patient 1, molecular genetic testing for CTX via *CYP27A1* Gene sequencing was recommended for Patient 2. The genetic test indicated that the patient is homozygous in *CYP27A1* gene for a sequence variant designated c.1016C > T, which is predicted to result in the amino acid substitution p.Thr339Met. Patient 2 was prescribed CDCA (15 mg/kg/day) and multivitamin drops (for vitamins A, D, E, and K).

As of March 2024, patient 2 is 7 years old without any signs of cataracts or neurological deficits. Diarrhea has improved. Even though her cognitive function was low, and her IQ was subnormal (IQ score was 72; normal average IG: 100); it was better compared to patient 1. For the past 8 months, patient 2 has been on combination therapy of CDCA and Sodium Bicarbonate (250 mg of CDCA with 25 cc of sodium bicarbonate), similar to patient 1. Her cognitive abilities demonstrated an improvement after the combination therapy. The last reported (March 2024) cholestanol levels were 23 mg/mL (Other cholestanol level data are unavailable due to resource limitations). Similar to Patient 1, she also has some dysmorphic features.

### 2.3 Patient 3

Patient 3 was a girl child, a sibling of patients 1 and 2, born full-term with a birth weight of 2.3 kg. There were no complications during pregnancy, and she was delivered via spontaneous vaginal delivery. Patient 3 was breastfed. Jaundice was observed on the third day of the life. This was accompanied by watery and yellowish feces with some oil; without any acholic stools and a bowel frequency of 3–4 times per day (Vesikari score: 5).

Patient 3 first visited the clinic at the age of one week. During this time, her total and direct bilirubin levels were 210 μmol/L and 15 μmol/L respectively. The liver enzyme tests showed normal levels for ALT (25 IU/L) and AST (10 IU/L). Repeat tests after one week for total and direct bilirubin showed the values of 155 μmol/L and 14 μmol/L respectively. Laboratory blood investigation reported PT of 12 s (normal range: 11 to 13.5 s), INR of 1.1 (normal range: 0.8 to 1.1); and PTT of 35 s (normal range: 33.3 to 56 s). Despite improvement in jaundice without any treatment, patient 3 was not thriving and diarrhea persisted.

At four months old, patient 3 presented to the clinic with chief concerns of failure to thrive and chronic diarrhea since birth. Physical examination showed that patient 3 was alert and active, able to control her head and smile. Patient 3 had subtle dysmorphic features such as a bulbous nose, prominent cheeks, and sparse hair, similar to her siblings. She was severely malnourished, with a weight of 4.2 kg (z score − 2.77) and a height of 52 cm (z-score: −3.83). Jaundice was not present, the liver was palpable at 2–3 cm from the costal edge, and the spleen was just palpable. Cardiovascular and neurological systems were normal.

Blood tests showed normal levels of total bilirubin (13 μmol/L), direct bilirubin (8.79 μmol/l), transaminases (ALT: 25 IU/L; AST: 28 IU/L), GGT (16 IU/L), and vitamin A (1.4 μmol/l). However, levels for vitamin D 25-OH (14 nmol/l), and vitamin E (3.4 μmol/l) were low. Elevated alkaline phosphatase (ALP) levels (1200 IU/L; normal range for a child up to 12 months: 150–240 IU/L) and cholesterol levels of 3.9 mmol/L were reported. Triglycerides levels were normal (1.3 mmol/L). Laboratory blood investigation revealed a prolonged activated partial thromboplastin time (aPTT) of 83 s (normal range: 30–40 s) and an INR of 7.2 (normal: 0.8–1.1). Repeat tests confirmed prolonged aPTT of 81 s and an INR of 7.5. There was no purpura or bleeding from any orifices. She received 4 mg of vitamin K, intravenously and the coagulation markers immediately improved (aPTT 18 s and INR 1.15).

Considering the clinical findings, laboratory findings, and family history, we immediately started a combination of CDCA (15 mg/kg/day) with sodium bicarbonate (250 mg of CDCA to be mixed with 25 cc of sodium bicarbonate). Along with this, multivitamin drops (for vitamins A, D, E, and K) were recommended and the mother was advised to continue breastfeeding.

After one week, diarrhea was improved; after two weeks, the stool frequency was once daily with a formed stool. Patient 3 began to grow and gained approximately 700 g in the first month after treatment. The molecular genetic testing by Single Gene using NGS for *CYP27A1* was performed. The test reported that the patient is homozygous for the pathogenic variant [NM_000784.3:c.1016C>T p.(Thr339Met)] in *CYP27A1* gene. As of March 2024, Patient 3 is 16 months old, with normal cognitive functions, no developmental delay, and absence of cataracts or any neurologic deficits.

## 3 Discussion

CTX is a rare disease with the uppermost prevalence in Asians, lowermost in Finnish, and intermediate in Europeans, Americans, and Africans/African Americans, with the ratio of 1:44,407–93,084, 1:3,388,767 and 1:70,795–233,597, respectively [6]. In line with these estimates, CTX is also considered rare in the Middle East, with limited case reports available. In previously published case studies, commonly reported symptoms included (Table 1) recurrent episodes of diarrhea, cataracts, Achilles tendon xanthomas, atherosclerosis, neurological symptoms, and unsteady gait [5,8,9,[13], [14], [15], [16]]. This table highlights a few case studies from Saudi Arabia indicating that CTX presents with both neurologic and extra-neurologic symptoms. In this case report, the diagnosis was suspected solely based on congenital diarrhea. However, there have been studies where diarrhea was not reported, highlighting the heterogeneity of the symptoms associated with CTX.

**Table 1:** Common symptoms of CTX observed in case studies.

Case studies of CTX from	Common symptoms reported	Reference number
Saudi Arabia	Chronic childhood diarrhea, premature cataracts, xanthomas of the Achilles tendons, neuropsychiatric disturbances, ataxia, atherosclerosis	[5]
Dutch	Diarrhea	[8]
Turkey	Cataracts, seizure, persistent diarrhea, intellectual disability, ataxia, developmental delay, xanthomas, tremor, jaundice, psychiatric manifestations,	[9]
Saudi Arabia	Cataracts, diarrhea, pain in shins, personality problem	[13]
Saudi Arabia	Cataracts, diarrhea	[14]
UK	Unsteady gait, swollen Achilles tendon, cataracts	[15]
Israel	Atherosclerosis, tendon xanthomas, cataracts, dementia	[16]

This case series documents CTX diagnosis in the youngest known patients, with chronic diarrhea as the primary presenting symptom prior to the onset of neurological or cataract manifestations. Patient 1, the index patient, underwent a diagnostic journey that involved numerous tests and spanned nearly 18 months before CTX was identified. However, recognizing persistent diarrhea as a symptom consistent with CTX, allowed for a targeted diagnostic approach in the siblings, significantly reducing the diagnostic timeline to under a month.

In this case series, persistent childhood diarrhea was the key clinical symptom leading to CTX diagnosis. Unfortunately, in previously reported CTX cases, persistent childhood diarrhea was discovered retrospectively [1,4,5,8,9,11,13,14,17]. The case study reported by A. O. Khan et al., (2014), reported that all patients diagnosed with CTX exhibited diarrhea during early childhood, even when the initial diagnosis was based on other indicators, such as – an unusual pattern of flecked lenticular deposits [13,14]. Hence, pediatric gastroenterologists must look for CTX when there is early-onset diarrhea, specifically, when accompanied by a history of infantile hepatic dysfunction. In our case reports, all three patients had childhood diarrhea and two of them also had a history of jaundice (Patients #1 and #3).

Treatment with CDCA, the current standard of therapy, led to significant improvements in diarrhea in all three patients. CDCA helps in restoring normal levels of sterol, bile acids, bile alcohol, and cholestanol. CDCA can also prevent adverse clinical manifestations of the disease from occurring or progressing, especially when administered early in the treatment [1,3,18,19]. The improvement of diarrhea in all three siblings supports the use of CDCA for CTX treatment. Patients have also been prescribed fat-soluble vitamin drops (Vitamins A, D, E, and K) to support fat malabsorption and diarrhea. There is substantial evidence that early treatment with CDCA can prevent the progression of the disease or its adverse clinical manifestations [3,4,[8], [9], [10],^13^,^17^,^18^,^20^].

Our patients are the youngest reported cases, where the CTX diagnosis was made at a very early age of 18 months (patient 1) and 4 months (patients 2 and 3). These are the first cases where CTX has been diagnosed at an early age of 4 months. As of December 2024, there are no reports of CTX being diagnosed at this early age. All three patients showed an improvement in diarrhea after treatment with CDCA. Currently, none of the three patients have cataracts or neurologic deficits. Initially, there was no improvement in cognitive functions, for patients 1 and 2, which could be due to non-compliance or issues with medication absorption. However, once the combination therapy of CDCA with sodium bicarbonate was initiated, the parents reported an improvement in awareness. This suggests that a better prognosis can be achieved with early diagnosis and long-term therapy with CDCA, which may improve the neurological symptoms.

In this study, certain dysmorphic features were visible in all three patients. These were the unique observations in the CTX patients and had not been previously reported. Further observations may be needed to confirm similar findings. Dysmorphic features should be assessed by physicians, as these findings can aid in a more accurate disease diagnosis. Vigilance for such features is especially important in patients presenting with symptoms related to CTX, as this could enhance diagnostic precision.

Considering the heterogeneity of the clinical manifestations of CTX, many times, the disease remains undiagnosed or misdiagnosed [7]. The diagnostic delay till the second or third decade of life is common for such patients. There is also patient inertia where families blame the “evil eye” or other superstitions for the disease as it also causes cognitive function decline [2].

This case study reinforces the need for heightened awareness among healthcare providers regarding the diverse presentations of CTX. This case study also highlights the common diagnostic delay, as it took nearly 18 months to diagnose CTX in Patient 1, the index patient. For his siblings, however, recognition of persistent diarrhea - an indicator for CTX - allowed for diagnosis in under a month. This underscores the importance of awareness among healthcare providers regarding CTX's diverse symptoms. Intractable childhood diarrhea, early cataracts, and/or relevant family history should be given a second thought, and detailed laboratory analysis should be performed to rule out or confirm the diagnosis of CTX.

In the current case series, diarrhea was the most common symptom that led to CTX diagnosis in children and infants. Initiation of CDCA treatment in combination with sodium bicarbonate resulted in an improvement in diarrhea with less stool frequency in all three patients. Due to timely diagnosis and treatment with CDCA, none of the siblings in our case series have developed cataracts or neurological deficits to date. A limitation of this study is the small sample size, which may limit generalizability. Future studies are needed to confirm these findings and explore the role of dysmorphic features in CTX diagnosis.

### 3.1 Clinical recommendations

This case series underscores the importance of recognizing persistent childhood diarrhea as a potential indicator of CTX, particularly when combined with a history of jaundice or infantile hepatic dysfunction. Pediatric gastroenterologists should consider CTX in differential diagnoses and pursue targeted diagnostic testing where appropriate. Early intervention with CDCA therapy, potentially combined with adjunct therapies such as sodium bicarbonate, can significantly improve patient outcomes and prevent disease progression.

## Ethics approval

The procedures used in this study adhere to the tenets of the Declaration of Helsinki. The study permission was granted by the Institution Research Board of King Fahad Medical City (IRB-KFMC) (publication No. 22–2550).

## Funding

No specific funding was received for this work.

## CRediT authorship contribution statement

**Badr Mohammad Alsaleem:** Writing – review & editing, Writing – original draft, Supervision, Project administration, Data curation. **Amna Basheer Ahmed:** Writing – review & editing, Writing – original draft, Data curation. **Muhannad M. Alruwaithi:** Writing – original draft, Data curation. **Tarig Yassin Alamery:** Writing – original draft, Data curation. **Norah Nasser Alrajhi:** Writing – original draft, Data curation.

## Declaration of competing interest

The authors declare that they have no known competing financial interests or personal relationships that could have appeared to influence the work reported in this paper.

## Data Availability

No data was used for the research described in the article.
